# Supplementary material for: Distinct molecular pathways mediate Mycn and Myc-regulated miR-17-92 microRNA action in Feingold syndrome mouse models
Source: Nat Commun. 2018 Apr 10;9:1352. doi: 10.1038/s41467-018-03788-7 (PMC5893605; doi:10.1038/s41467-018-03788-7)

SUPPLEMENTARY INFORMATION

Distinct molecular pathways mediate Mycn and Myc-regulated miR-17-92 microRNA action in Feingold syndrome mouse models

Mirzamohammadi et al.

Supplementary Figures

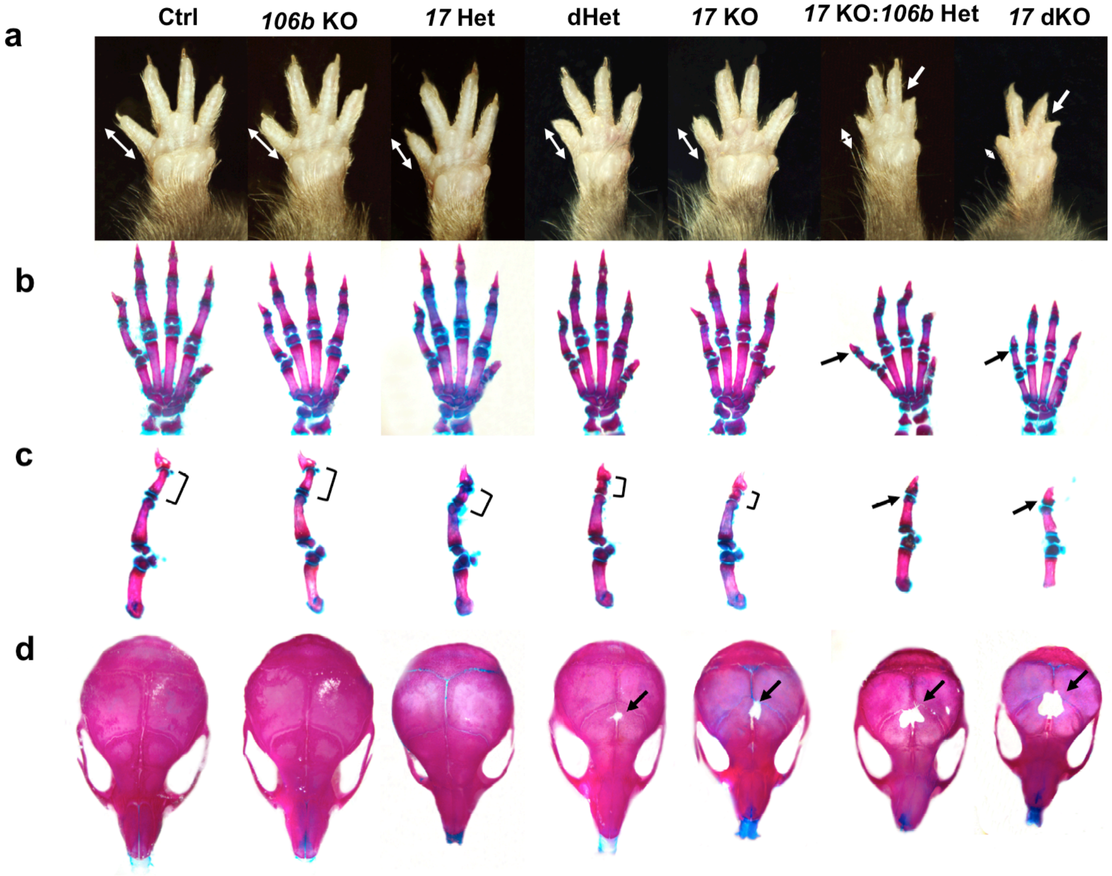

Supplementary Fig. 1. miR-17-92 and miR-106b-25 cluster miRNAs play overlapping roles in skeletal development. **a** Forelimbs of wildtype control (Ctrl) and mutant mice. *Mir106b-25* germline deletion (*Mir106b-25*$^{-/-}$,*106b* KO) causes no overt abnormalities. Conditional heterozygous or homozygous deletion of *Mir17-92* (*Prx1-Cre:Mir17-92*$^{fl/+}$*, 17* Het; and *Prx1-Cre:Mir17-92*$^{fl/fl}$*, 17* KO) and heterozygous deletion of both *Mir17-92* and *Mir106b-25* (*Prx1-Cre:Mir17-92*$^{fl/+}$*:Mir106b-25*$^{+/-}$, dHet) cause mild brachydactyly in the fifth digit (white double-headed arrows). Homozygous deletion of *Mir17-92* with heterozygous or homozygous deletion of *Mir106b-25* (*Prx1-Cre:Mir17-92*$^{fl/fl}$*:Mir106b-25*$^{+/-}$, *17 KO:106b* Het; and *Prx1-Cre:Mir17-92*$^{fl/fl}$*:Mir106b-25*$^{-/-}$, *17* dKO) exhibited severe brachydactyly and syndactyly (white single-headed arrows), phenotypes similar to those in patients with Feingold syndrome type 2. **b**, **c** Alizarin red and alcian blue staining of Ctrl and mutant forelimbs (***b***) and the fifth digit (***c***). Mutants show shortening of the middle phalanx (brackets) of the fifth digit. The middle phalanx of the fifth digit in *17 KO:106b* Het and *17* dKO mutants is absent (arrows). **d** Conditional deletion of *Mir17-92* and *Mir106b-25* causes microcephaly and ossification defects (arrows) with varying degree of severity depending on numbers of deleted alleles.

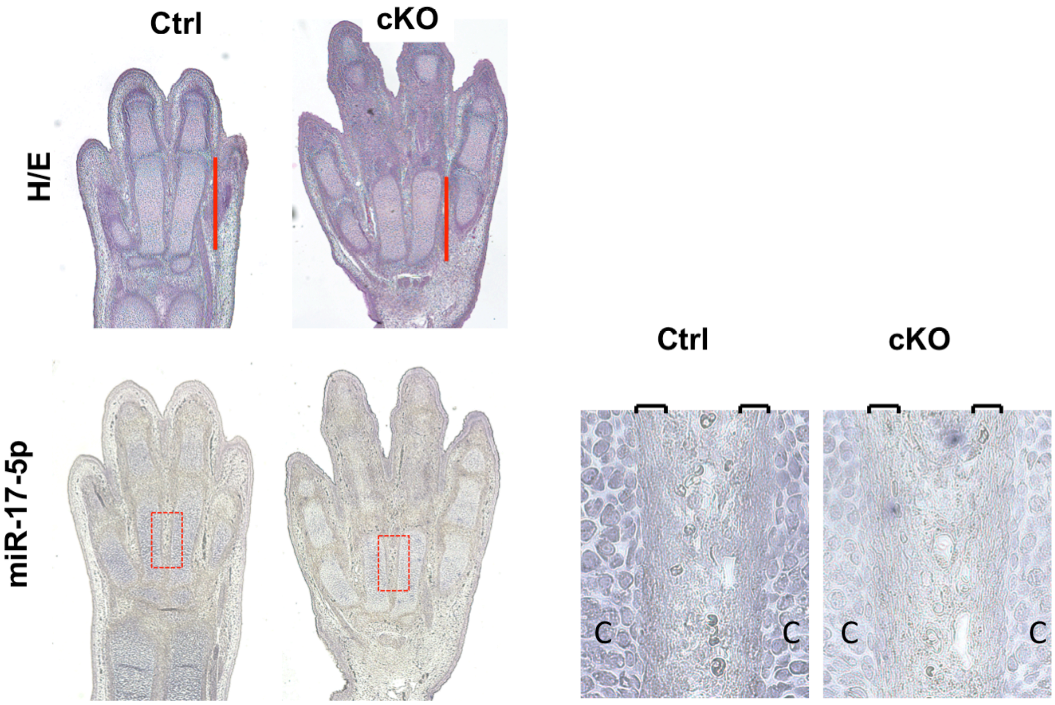

Supplementary Fig. 2. miR-17 expression in *Mycn*-deficient limbs at E14.5. *In situ* hybridization was performed to detect mature miR-17 (mmu-miR17-5p) in E14.5 forelimbs of Mycn conditional knockout mice (*Mycn* cKO; *Prx1-Cre:Mycn*$^{fl/fl}$)and their *Cre*-negative littermate control. ***Top panel***. Cartilage development of *Mycn*-deficient limbs is impaired. No significant alterations in the cellular makeup were found in Mync cKO paws. Red lines indicate the length of the fourth metacarpal bones. ***Bottom panels.*** miR-17-5p is expressed mainly in chondrocytes and perichondrial cells. The miR-17 expression is generally reduced in *Mycn*-deficient limbs. Magnified views of the areas indicated by dotted red lines in the left panel are shown in the right. Brackets indicate perichondrial layers. C indicates chondrocytes of metacarpal bones.

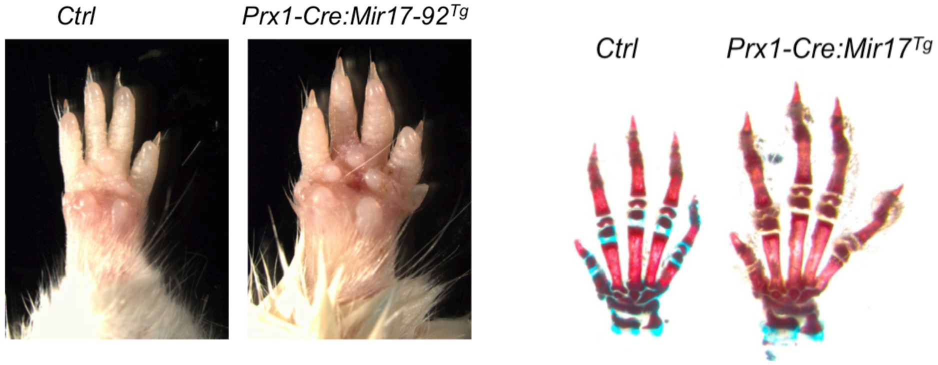

Supplementary Fig. 3. Limb overgrowth of mice expressing *Mir17-92 Tg* in limb mesenchyme. Representative pictures of P9.5 forelimb of *Prx1-Cre:Mir17-92*^{Tg} (*17* Tg) mice and littermate control**.**

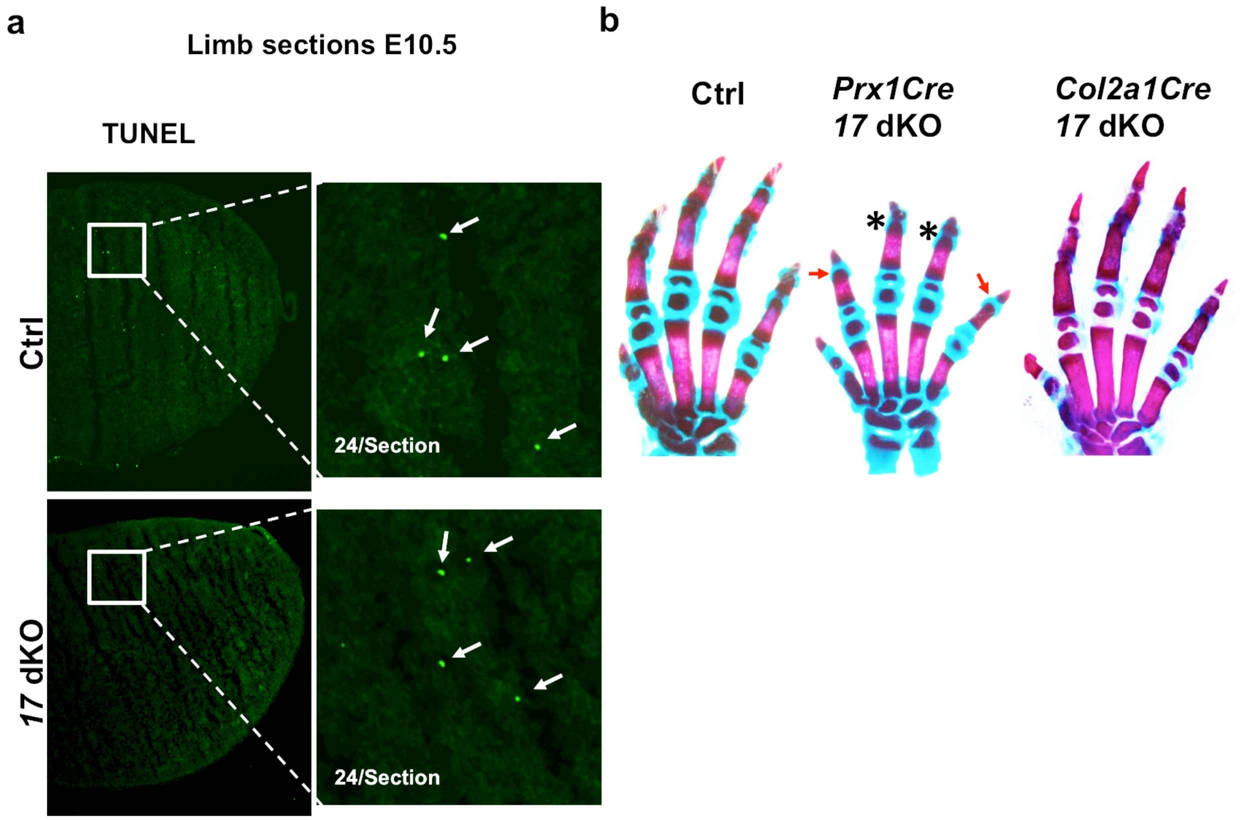

Supplementary Fig. 4. The skeletal phenotype of *Mir17-92:Mir106b-25* mutants is a consequence of an early developmental defect. **a** TUNEL Assay *In situ* cell death detection on limb bud sections of *Mir17-92*$^{fl/fl}$*:Mir106b-25*$^{-/-}$ (Ctrl) and *Prx1-Cre:Mir17-92*$^{fl/fl}$*:Mir106b-25*$^{-/-}$ (*17* dKO) embryos at embryonic day 10.5 (E10.5). *Mir17-92:Mir106b-25* deficiency does not increase cell death in the early limb bud. **b** Conditional deletion of *Mir17-92* and *Mir106b-25* in developing limbs at an early developmental stage using *Prx1-Cre* transgenic mice causes skeletal abnormalities in mice, whereas conditional deletion of *Mir17-92* and *Mir106b-25* exclusively in the cells of the precartilaginous condensation and their descendants using *Col2a1-Cre* transgenic mice caused no obvious skeletal abnormalities. Asterisks and red arrow indicate shortening and absence of the middle phalanx, respectively.

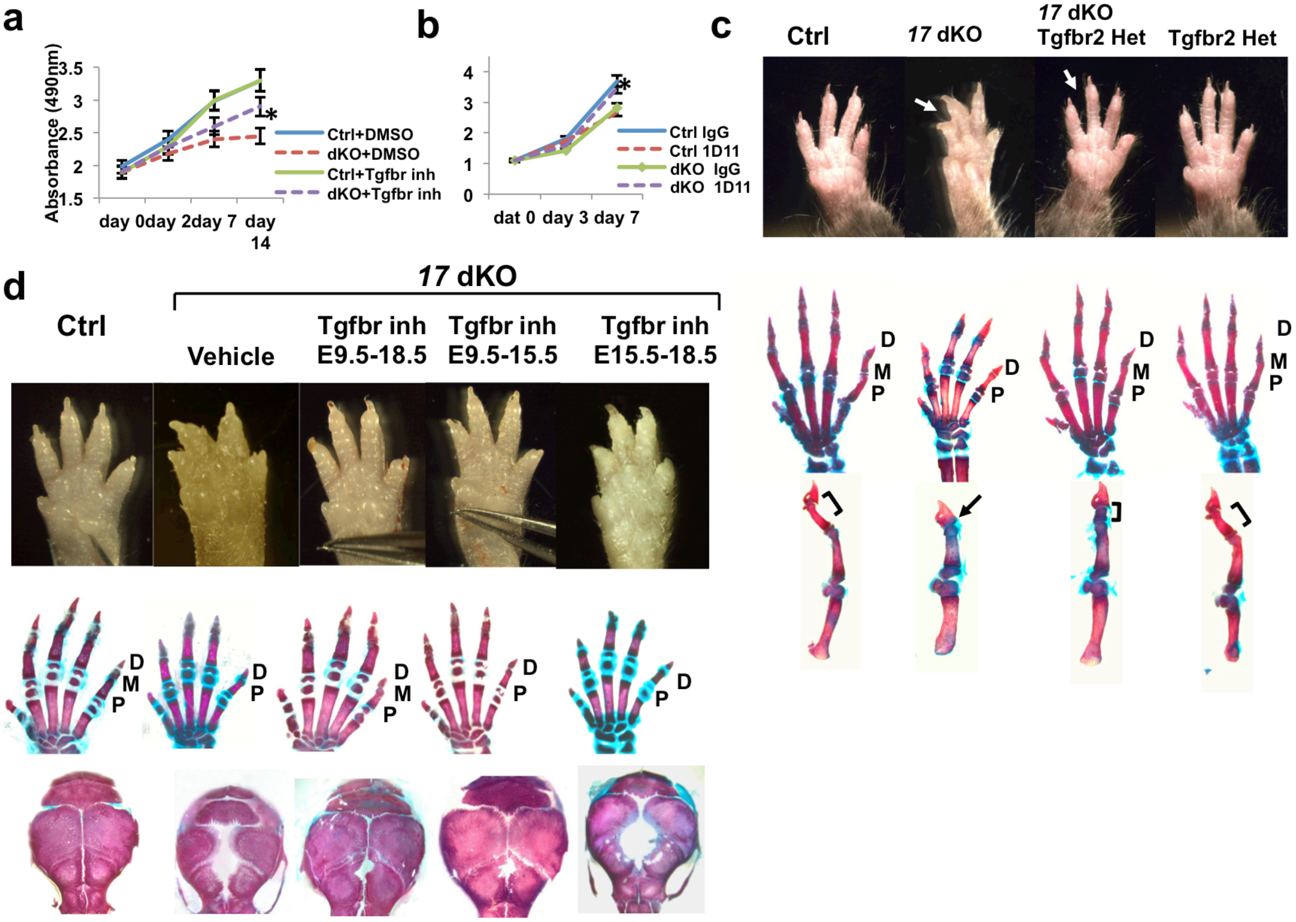

Supplementary Fig. 5. Effects of different treatment regimens of TGF-β inhibition. **a** Cell proliferation assay on control (Ctrl) and *Mir17-92:Mir106b-25*-deficient (*17* dKO) limb bud mesenchymal cells treated with vehicle (DMSO) or a TGF-β receptor inhibitor (Tgfbr inh, GW788388; 10 uM). Treatment with GW788388 had no significant effect on control cells but significantly ameliorated the proliferation defect of *17* dKO cells. (Values expressed as mean ± SE, n = 3, **p* < 0.05 vs dKO+DMSO). **b** Cell proliferation of Ctrl and *17* dKO cells treated with anti TGF-β 1-2-3 antibody (1D11, 5ug/ml) or IgG. Treatment with 1D11 significantly ameliorated the proliferation defect of *17* dKO cells (Values expressed as mean ± SE, n = 3, **p* < 0.05 vs dKO + IgG). **c** Skeletal phenotypes of wildtype control (Ctrl), *17* dKO, *17* dKO in which one allele of *Tgfbr2* is deleted (*Prx1-Cre:Mir17-92*$^{fl/fl}$*:Mir106b-25*$^{-/-}$*:Tgfbr2*$^{fl/+}$, *17* dKO:*Tgfbr2* Het), and *Tgfbr2* heterozygous (*Prx1-Cre:Tgfbr2*$^{fl/+}$, *Tgfbr2* Het) mice at the postnatal day 16. *Tgfbr2* heterozygous mutants have no obvious abnormalities, but deletion of one allele of *Tgfbr2* in *17* dKO mutants partially rescues skeletal defects of *17* dKO mutants including shortening of the fifth digit, syndactyly (white arrows), and the missing middle phalanx in *17* dKO mutants (M) (bottom panels). **d** Skeletal phenotypes of 9 day-old mice with TGF-β receptor inhibitor treatment. Treatment with the TGF-β receptor inhibitor, GW788388 (1 mg/kg/day), during only the embryonic stages (from E9.5 through E18.5) rescues skeletal defects of *17* dKO. Moreover, a short-term treatment regimen (E9.5-E15.5) mostly rescues the limb and skull abnormalities, whereas a late treatment regimen in which TGF-β receptor inhibitor injection is started at E15.5 shows little effect. D, distal phalanx; M, middle phalanx; P, proximal phalanx.

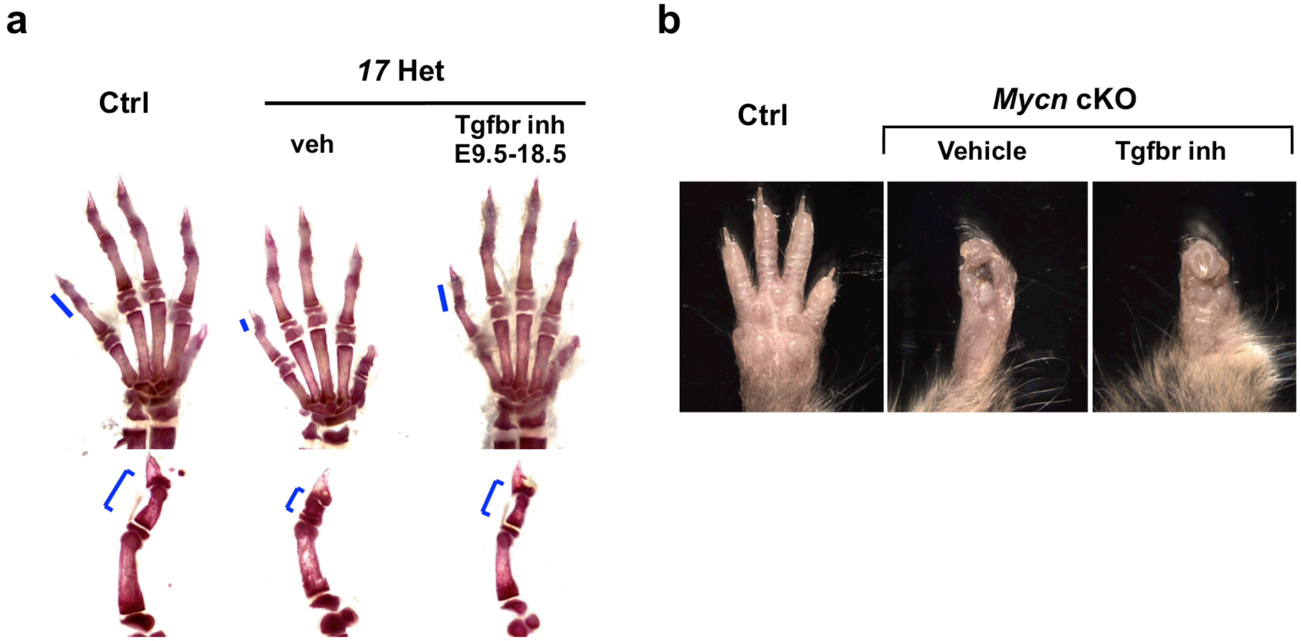

Supplementary Fig. 6. Effects of TGF-β inhibitor treatment in *Mir17-92* hetrozygous cKO and *Mycn* cKO. **a** Treatment with the TGF-β receptor inhibitor, LY364947 (Tgfbr inh, 1 mg/kg/day, i.p.) from E9.5 to E18.5 rescued the diminished middle phalanx of the fifth digit (indicated by blue brackets and bars) of *Mir17-92* conditional heterozygous knockout mice (*17 Het, Prx1-Cre:Mir17-92*$^{fl/+}$). **b** LY364947 (Tgfbr inh) injection did not rescue the digit phenotype of Mycn conditional knockout mice (*Mycn* cKO)

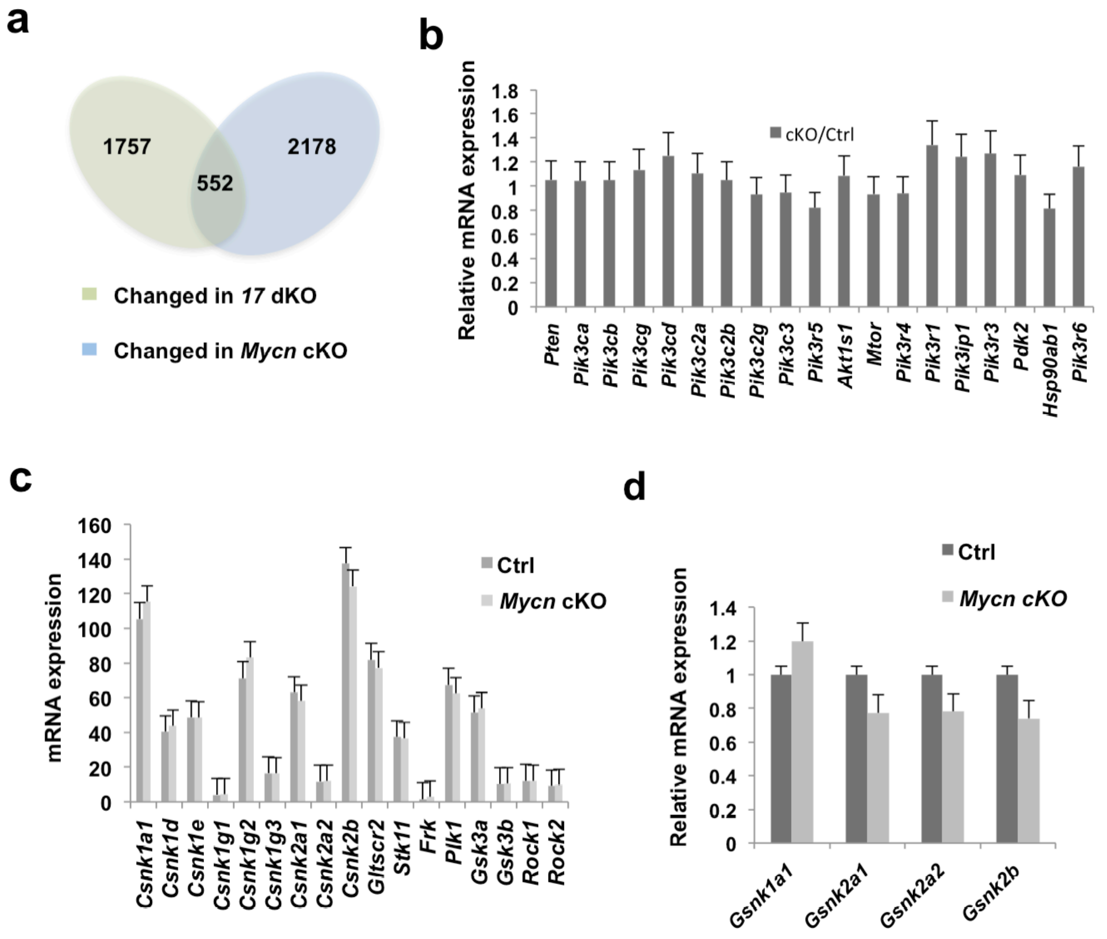

Supplementary Fig. 7. Gene expression profiles of *Mycn*-deficient and *17* dKO limb mesenchymal cells. **a** Venn diagram summarizing the overlap in the number of genes deregulated in E 10.5 *Mycn*-deficient and *Mir17-92*-deficient limb bud fibroblasts (RNA-seq data, fold change > 1.5 based on average values of three samples per group). There is a limited overlap in the transcriptome between *Mycn*-deficient and *Mir17-92*-deficient limb bud cells. **b** Relative mRNA expression of genes involved in the PI3K signaling pathway extracted from RNA-Seq data. The expression level of genes related to the PI3K signaling pathway is not altered in *Mycn*-deficient cells. **c** Expression levels of genes encoding kinases known to regulate phosphorylation of Pten. There are not significant changes in the expression level of casein kinase 2 beta (*Gsnk2a1*, *Gsnk2a2, Gsnk2b)* and other kinase-related genes. **d** Independent confirmation of the RNA-seq results of *c* by qRT-PCR analysis. There are no significant changes in expression of casein kinase 1 and 2 genes in *Mycn* cKO limb bud mesenchymal cells compared with wildtype control (n = 3, $p < 0.05$). Data are expressed as mean ± SEM.

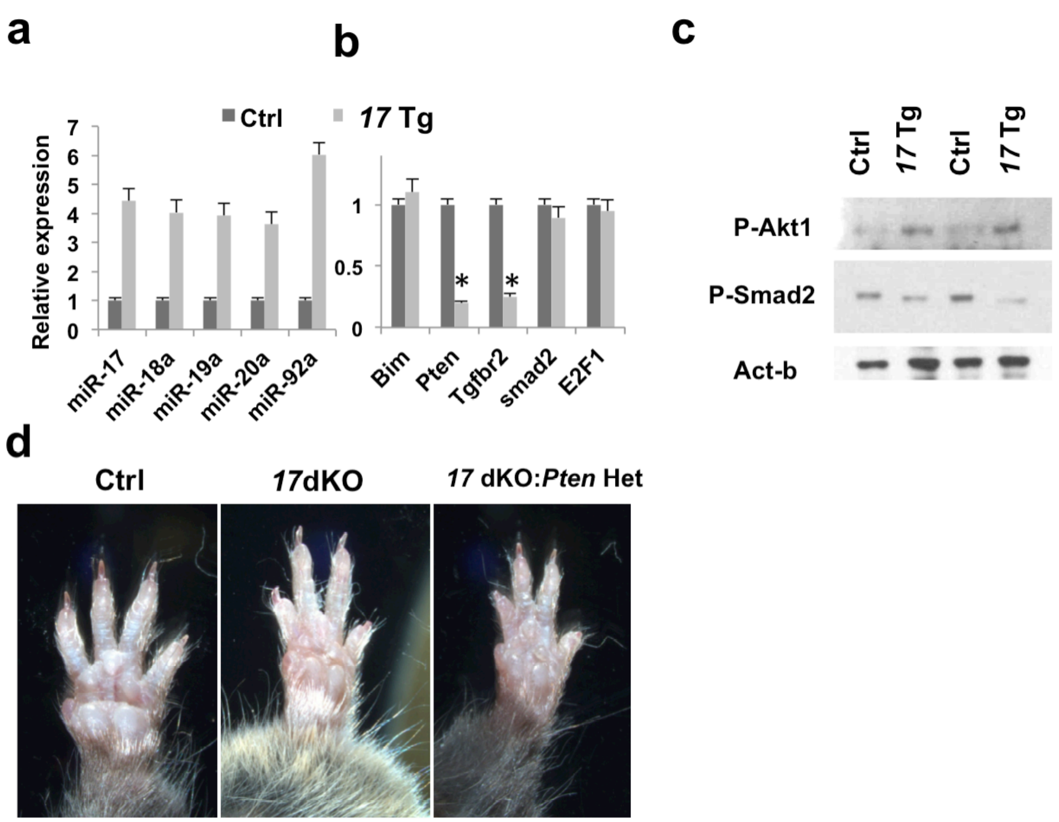

Supplementary Fig. 8. Overexpression of *Mir17-92* suppresses *Pten* and *Tgfbr2*. a Relative expression of miR-17-92 miRNAs in limb bud cells isolated from *Mir17-92* transgenic mice (*Prx1-Cre:Mir17*-92^{Tg} , *17* Tg). *CAG* promoter-driven *Mir17-92* overexpression causes four-fold increases in miR-17-92 miRNA expression in E10.5 limb bud cells compared with wildtype littermate control (Ctrl) (data expressed as mean ± SEM, n = 3, * $p < 0.05$). **b** Relative mRNA expression levels of known miR-17-92 targets in *17* Tg cells at E10.5. (data expressed as mean ± SEM, n = 3, * $p < 0.05$). The supraphysiological levels of miR-17-92 miRNAs in *17* Tg limb mesenchymal cells can suppress *Pten* in addition to *Tgfbr2*. **c** *Mir17-92* overexpression decreases p-Smad2 and increases p-Akt, indicating downregulation of TGF-β signaling and upregulation of PI3K signaling, respectively. **d** Deletion of one allele of *Pten* in *17* dKO does not rescue the skeletal defects of *17* dKO mutant mice.

Supplementary Fig. 9
Uncropped blots

Fig. 3.

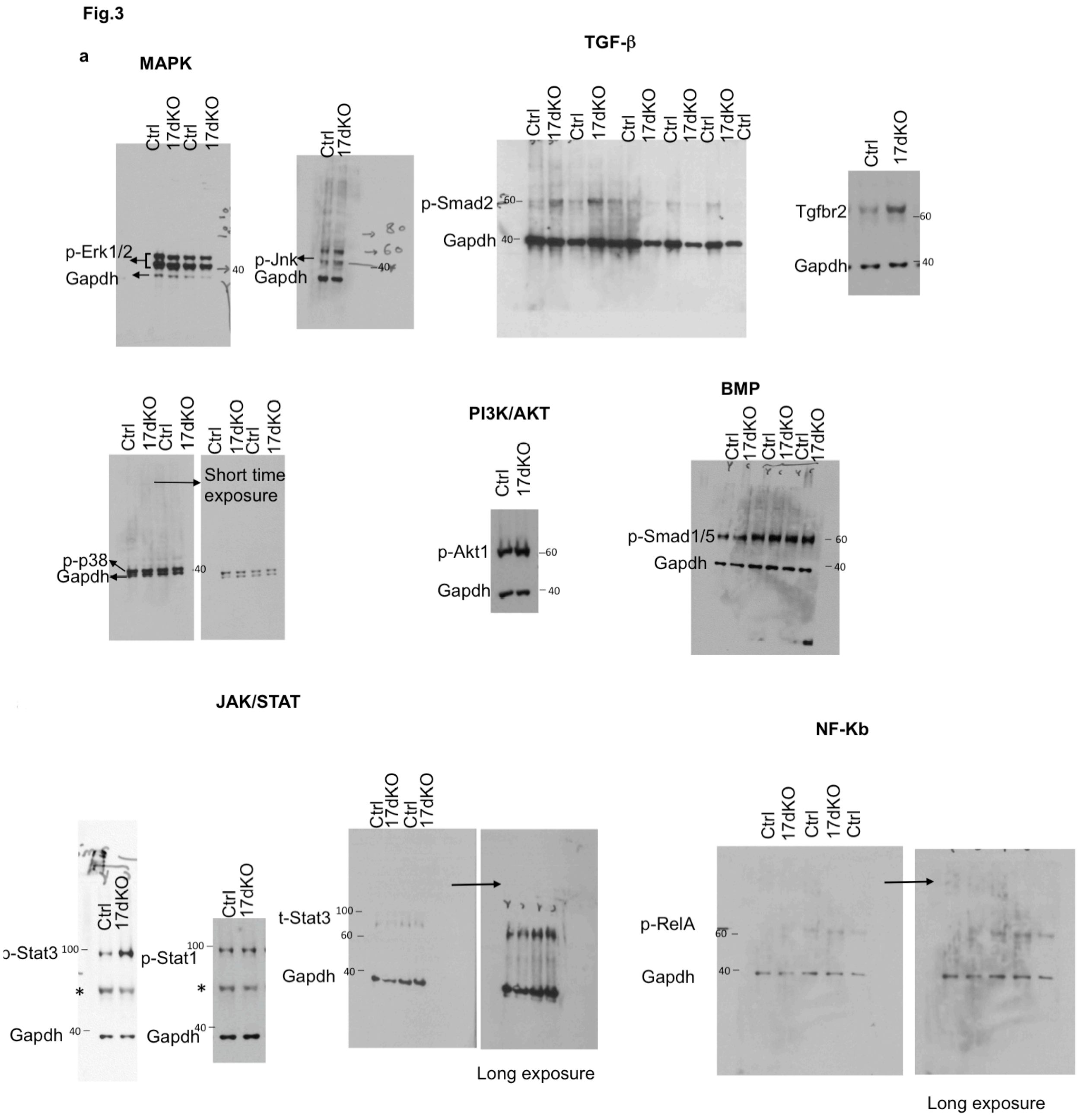

Supplementary Fig. 10
Uncropped blots

Fig. 3 (cont.)

Fig. 3

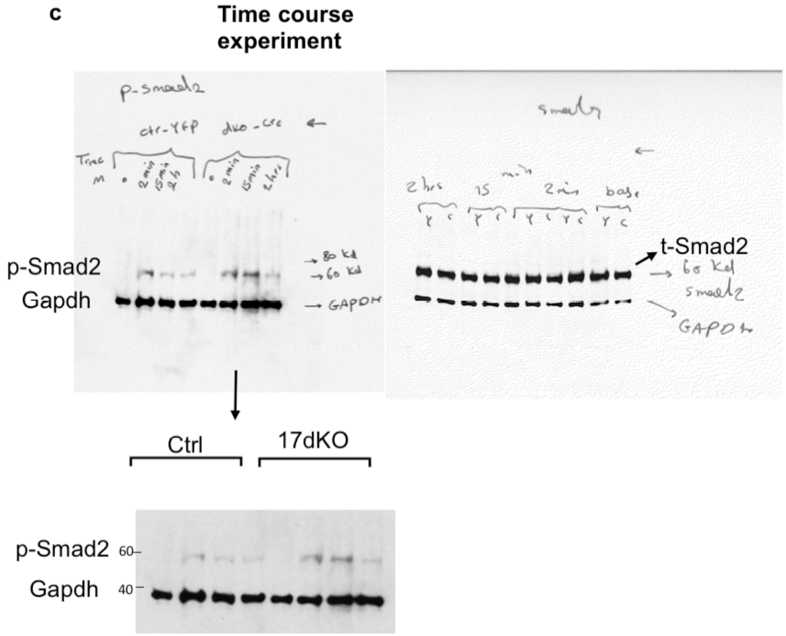

Fig. 4

Fig. 4

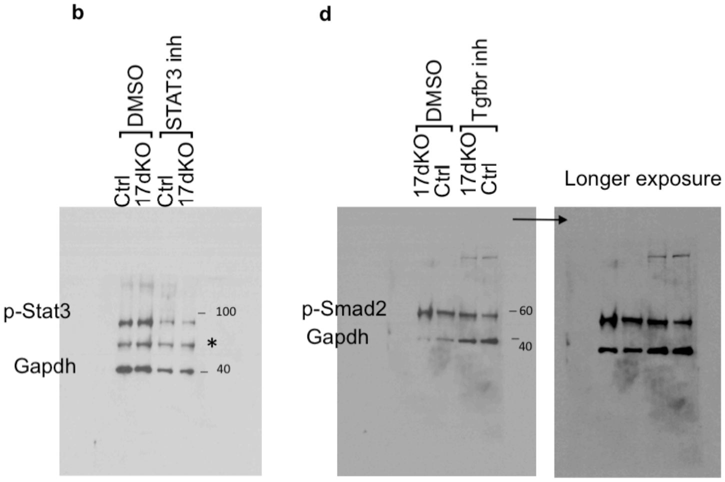

Supplementary Fig. 11
Uncropped blots

Fig. 5

Fig. 5

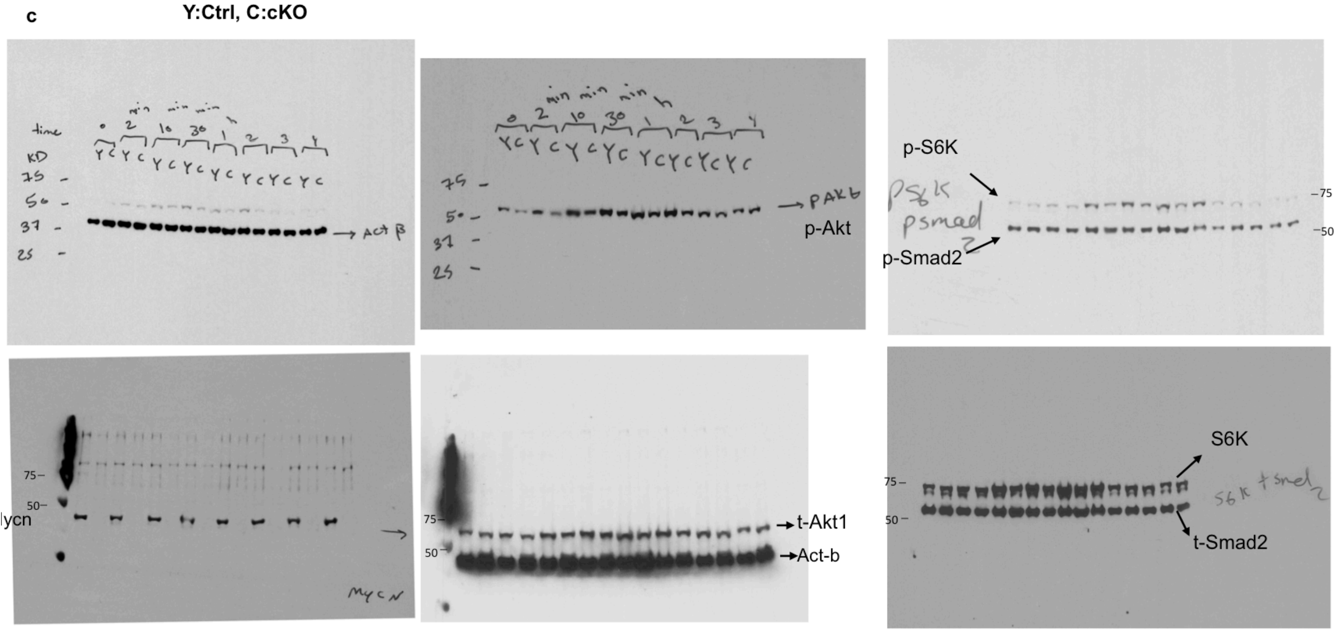

Fig. 6

Fig. 6

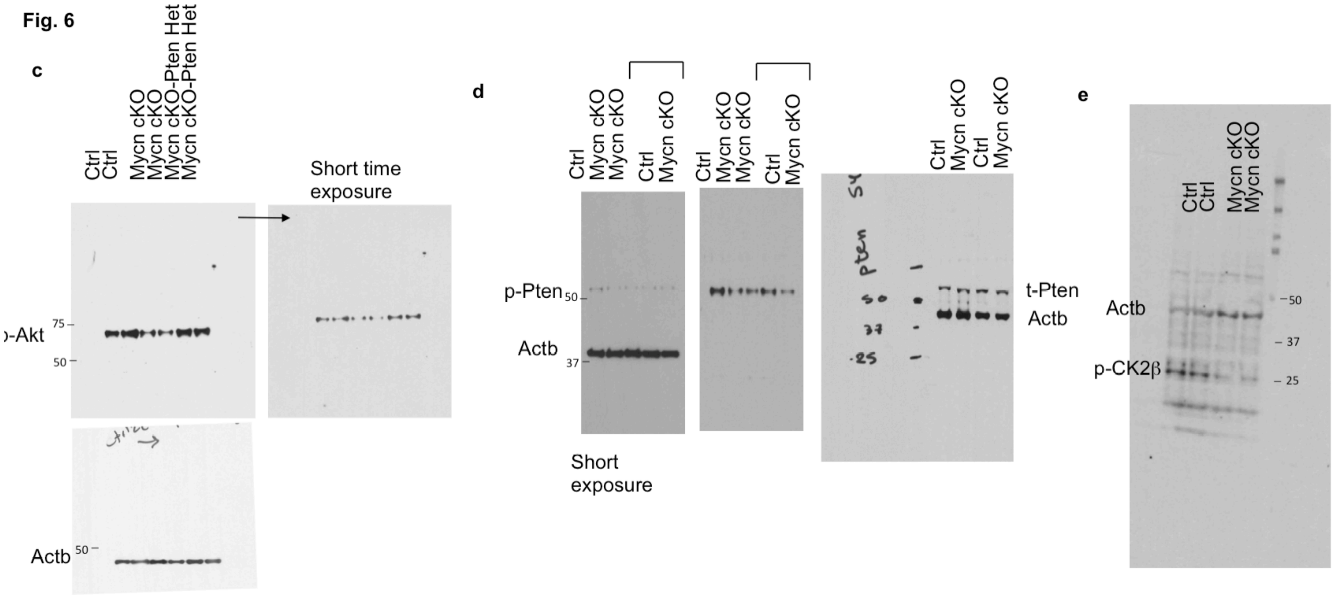

Supplementary Fig. 12
Uncropped blots

Supplementary Fig. 8

Supplementary Fig. 8 c

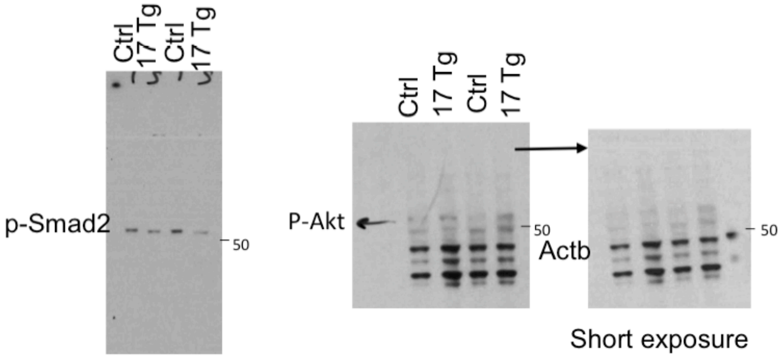

Supplement: Supplementary file 1 — Supplementary Information [file 41467_2018_3788_MOESM1_ESM.pdf]
