# Supplementary material for: Distinct molecular pathways mediate Mycn and Myc-regulated miR-17-92 microRNA action in Feingold syndrome mouse models
Source: Nat Commun. 2018 Apr 10;9:1352. doi: 10.1038/s41467-018-03788-7 (PMC5893605; doi:10.1038/s41467-018-03788-7)
Supplement: Supplementary file 3 — Description of Additional Supplementary Files [file 41467_2018_3788_MOESM3_ESM.docx]

**Description of Additional Supplementary Files**

File Name: Supplementary Data 1

Description: Gene expression profiles of Mycn- and Mir-17-92:Mir106b-deficient limb buds
